# Synthesis of Sulfonated Trimer Peptides to Discover Potential Drug Candidates Targeting Amyloid‐β in Alzheimer's Disease

**DOI:** 10.1002/alz70861_108192

**Published:** 2025-12-23

**Authors:** Sunghyun Kim, Minhae Cha, YoungSoo Kim

**Affiliations:** ^1^ Yonsei University, Incheon, Incheon Korea, Republic of (South); ^2^ Yonsei Institute of Pharmaceutical Sciences, Incheon, Incheon Korea, Republic of (South); ^3^ Amyloid Solution Inc., Seongnam, Gyeonggi‐do Korea, Republic of (South); ^4^ College of Pharmacy, Yonsei University, Incheon, Incheon Korea, Republic of (South); ^5^ Yonsei University, Yeonsu‐gu, Incheon Korea, Republic of (South)

## Abstract

**Background:**

The accumulation of amyloid‐β (Aβ) in the form of neurotoxic aggregates is widely recognized as a central pathological hallmark in Alzheimer’s disease (AD). Recent progress in immunotherapeutic strategies has validated the efficacy of Aβ clearance in treating Alzheimer’s disease, thereby establishing Aβ deposits as a promising target for therapeutic intervention. Given the numerous reports emphasizing the importance of sulfonic acid group in targeting Aβ, we aim to synthesize sulfonated trimer peptides for later evaluation of therapeutic efficacy.

**Method:**

Trimer peptides were synthesized via standard Fmoc‐based solid‐phase peptide synthesis (SPPS). After the completion of synthesis of trimer, global deprotection and cleavage from the resin were performed using trifluoroacetic acid (TFA) to release the peptides. Following peptide synthesis, O‐sulfonation of the serine hydroxyl group was carried out using chlorosulfonic acid as the sulfonating agent. Then, the sulfonated trimer peptides were purified using reverse‐phase high‐performance liquid chromatography (RP‐HPLC).

**Result:**

A total of 27 sulfonated trimer peptides were rationally designed and successfully synthesized. The library included trimers containing sulfonated serine at different positions. The peptides were synthesized with an average crude yield of approximately 50%. Subsequently, all peptides were purified using reverse‐phase high‐performance liquid chromatography (RP‐HPLC), yielding final products with an average purity of approximately 90%.

**Conclusion:**

As a result, a total of 27 sulfonated trimer peptides were successfully synthesized and purified. Through subsequent efficacy validation of the synthesized trimer peptides, the essential contribution of sulfonation to amyloid‐β targeting will be elucidated.

